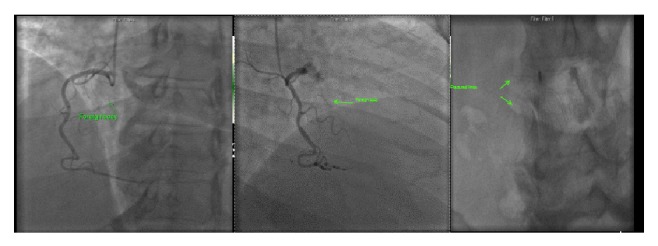# Wire in the Heart: Fracture and Fragment Embolization of Retrievable Inferior Vena Cava Filter into the Right Ventricle

**DOI:** 10.1155/2015/938184

**Published:** 2015-04-21

**Authors:** Kshitij Thakur, Naveen Dhawan, Chia Winchester, Amandeep Singh, Vijay Bodukam, Jaya Bahl

**Affiliations:** ^1^Crozer-Chester Medical Center, Upland, PA 19081, USA; ^2^Nova Southeastern University Health Sciences Division, Fort Lauderdale, FL 33314, USA; ^3^Florida International University (FIU), North Miami Beach, FL 33199, USA

## Abstract

We report a case of a 58-year-old female who was found to have a fractured limb of her IVC filter in her right ventricle during a cardiac catheterization. A 25 mm radioopaque thin linear structure was seen in the proximal portion of the right ventricle. It was fixed and did not migrate or change position during investigations. On fluoroscopy, the IVC filter was observed in an appropriate location in the midabdomen. Yet, fractures of at least two of the metal filamentous legs of the IVC device were noticed. The patient was made aware of the many risks associated with filter removal. Due to the high risks of the procedure, she refused surgery and the filter fragment was not removed. We present this case to underscore the potential complications of IVC filters.

## 1. Introduction

Deep vein thrombosis (DVT) is a potentially fatal condition with over 200,000 cases per year [1]. The routine therapy for patients with DVT has been anticoagulation [2]. The American College of Chest Physicians recommends the use of an inferior vena cava (IVC) filter in patients with acute proximal DVT of the leg and with contraindications to anticoagulation [3]. In recent years, there has been an increase in the use of such filters, although it appears that a majority of filters are not removed [4]. IVC filters provide a screen in the inferior vena cava, allowing blood to pass while blocking the passage of large emboli from the pelvis or lower extremities before reaching the lungs. IVC filters can be divided into retrievable and nonretrievable filters. Retrievable filters were developed to reduce the long-term complications associated with permanent filters, in particular, the increased risk of DVT. Up to 70% of retrievable filters are never removed.

Few cases of fractured IVC filter limb migration to the heart, pulmonary veins, and the renal vein have been previously reported. The case of a IVC filter embolization directly to the heart in a 55-year-old male was recently described [5]. We report a case of a 58-year-old female who was found to have a fractured limb of her IVC filter in her right ventricle during a cardiac catheterization.

## 2. Case Presentation

A 58-year-old African American patient presented to our hospital with complaints of dyspnea on exertion and intermittent chest discomfort for 2-3 months. Her past history was remarkable for hypertension, diabetes mellitus, DVT, and PE status after retrievable IVC filter placement in 2005 due to failure of anticoagulation. At the time of presentation, she reported smoking half pack per day with cocaine use in recent times. On physical exam, there were no significant findings; S1 and S2 sounds were normal. There were no murmurs, rubs, or gallops. Both lungs were clear to auscultation. After the initial presentation to her PCP, she was referred to a cardiologist for a stress test which showed signs of ischemia. A cardiac catheterization was recommended after this abnormal stress test.

A 25 mm radioopaque thin linear structure was seen moving with cardiac motion in the region of the proximal portion of the right ventricle (Figure 1). This object was fixed and did not migrate or change position during the study. On fluoroscopy, the IVC filter was seen to be in appropriate location in the midabdomen. However, fractures of at least two of the metal filamentous legs of the device were visible. The patient was made aware of the risks of filter removal. Due to the high risks of the procedure, she refused surgery and the filter fragment was left in situ.

## 3. Discussion

Retrievable IVC filters are routinely used to decrease the occurrence of pulmonary embolism, particularly in patients with contraindications to anticoagulants [6]. They are also used to avert bleeding from anticoagulation given systemically [7]. Yet, the safety of these filters has been questioned following reports in the literature of fracture fragment embolization, even leading the Food and Drug Administration (FDA) to put forth a warning pertaining to retrievable filters in 2010 [6]. It has been proposed that IVC filter fracture and resulting embolization occur in up to 25% of patients on Bard retrievable IVC filters, leading to adverse cardiac episodes including ventricular arrhythmia and cardiac tamponade [2]. The migration of IVC filters of over 1 cm in distance has been reported in up to 5% of cases [8].

Complications of IVC filters include local complications related to the insertion process, DVT at the site of insertion, filter migration and erosion, cardiac tamponade, perforation of right ventricle, fracture and fragment embolization, and IVC thrombosis/obstruction [2]. Importantly, mortality from filter placement is low, approximately 0.12–0.3%.

Patients with migration of fractures to the right heart may present with the same symptoms of pulmonary embolus: shortness of breath, pleuritic chest pain, discomfort, and syncope. Thus, it is important that physicians have knowledge of the complications of IC filter fracture since some patients who might subsequently be seen with presumed symptoms of recurrent PE may have had filter fracture, migration, and perforation, necessitating a different course of intervention. Some factors implicated in filter fracture include filter design (greater penetration and thus stress are reported more in short limbs than in long limbs) and the comprising material [6].

Importantly, certain filters seem to be more promising in their low rates of adverse effects than others. One retrospective review of 741 IVC filter implantations reported, for example, that the Celect filters are largely resistant to the development of fracture [6]. Yet, in another study, Bard Recovery filters showed fracture in 25% of cases (12% in Bard G2 filters), although these were based on lengthy durations from implantation to filter assessment; the incidence of fracture appears to increase with filter dwelling time [2].

It is essential that patients and their physicians be educated about the underrecognized and potentially life-threatening complications of IVC filters. Additionally, an emphasis should be placed on prompt removal of a retrievable filter when there is no longer a need for protection from pulmonary embolus. A major challenge occurs in the decision to remove the filters. Our patient refused filter retrieval due to the many known risks that were explained to her. Future studies that closely examine the factors associated with IVC filter fracture following implantation are warranted. Further investigations of which specific filter types are more prone to fracture can help clinicians in their decisions to implant IVC filters.

## Figures and Tables

**Figure 1:**